# Hypersensitivity reactions amongst Hungarian Patients with Hereditary Angioedema due to C1-Inhibitor Deficiency

**DOI:** 10.1016/j.waojou.2023.100833

**Published:** 2023-10-23

**Authors:** Hanga Réka Horváth, Dávid Szilágyi, Noémi Andrási, Zsuzsanna Balla, Beáta Visy, Henriette Farkas

**Affiliations:** a Hungarian Angioedema Center of Reference and Excellence, Department of Internal Medicine and Haematology, Semmelweis University, Budapest, Hungary; b Doctoral School, Semmelweis University, Budapest, Hungary; c Pediatric Center, Tűzoltó Street Department, Semmelweis University, Budapest, Hungary; d HNO-Praxis Schaffhausen, Schaffhausen, Switzerland; e Heim Pál National Institute of Pediatrics, Budapest, Hungary

**Keywords:** Allergic diseases, Bradykinin, Heparin, Hereditary angioedema due to C1-inhibitor deficiency (C1-INH-HAE), Hypersensitivity

## Abstract

**Background:**

In hereditary angioedema (HAE) due to C1-inhibitor deficiency (C1-INH-HAE), bradykinin-mediated submucosal and/or subcutaneous angioedema dominates the clinical picture. The deficiency of C1-inhibitor can lead to the over-activation of the complement system. Complement plays an important role in all types of hypersensitivity reactions. On the other hand, during the degranulation of mast cells, heparin is also released amongst other substances. Heparin can activate the plasma kinin-kallikrein system, leading to bradykinin generation. These observations suggest a possible connection between C1-INH-HAE and mast cell-mediated hypersensitivity reactions.

**Objective:**

To assess the occurrence of hypersensitivity reactions in the Hungarian C1-INH-HAE population.

**Methods:**

Patients filled out a questionnaire of 112 questions, either online or on paper. The questions were about hypersensitivity and C1-INH-HAE symptoms, the relation between these 2, general health, and demographic data. The study protocol was approved by the institutional review board of Semmelweis University, Budapest, and informed consent was obtained from the participants.

**Results:**

One hundred and six patients (64 female, 42 male, median age 46 years) responded, with 63.2% having hypersensitivity. Hypersensitivity was provoked by pollen in 25.5% of patients, by contact sensitivity in 22.6%, by food in 21.7%, by insect sting in 19.8%, by pet in 15.1%, by drug in 14.2%, by dust mite in 5.7%, and by mold in 1.9%. In 11 patients, hypersensitivity symptoms appeared after the diagnosis of C1-INH-HAE. Six hypersensitive patients experienced improvement in their symptoms; 42 remained the same, but none experienced worsening after the diagnosis of C1-INH-HAE. In 7.8% of the hypersensitive patients, a C1-INH-HAE attack worsened the hypersensitivity symptoms, while 15.7% of the hypersensitive patients experienced a C1-INH-HAE attack provoked by contact with the provoking factor.

**Conclusion:**

While 63.2% of our C1-INH-HAE patients have reported hypersensitivity symptoms, Eurostat's latest data puts the prevalence of self-reported allergies in Hungary at 19.3%. Since in our experience most Hungarian patients report hypersensitivity reactions as allergies, this may support a possible connection between the 2 diseases, but further molecular studies are needed.

## Introduction

Hereditary angioedema (HAE) is a rare, autosomal dominantly inherited disease characterized by recurrent subcutaneous and/or submucosal swellings (HAE attacks). The angioedema is not associated with wheals or itching and does not respond to the conventional antihistamine, corticosteroid, or adrenaline treatment.^1^^,^^2^ Subcutaneous HAE attacks cause deformity and dysfunction of the extremities or the face, that leads to missing school or work and can cause severe psychosocial stress. Submucosal HAE attacks may appear in the upper airways or in the intestinal tract. Laryngeal attacks are life-threatening because they can cause suffocation and, in some cases, lead to intubation and/or tracheostomy that could have been avoided with appropriate treatment. Intestinal HAE attacks can mimic an “acute abdomen”, and, as such, can lead to surgical procedures such as appendectomy. The site, frequency, and severity of HAE attack occurrence has high intra- and interindividual variability.^3^^,^^4^

In its most common form, HAE is caused by the reduced production (type I) or dysfunction (type II) of the C1 inhibitor protein (C1-INH).^5^^,^^6^ C1-INH is the regulator of many immunological pathways, including the classical and lectin pathways of the complement system, the contact activation system, the fibrinolytic pathway, and the plasma kinin-kallikrein system (PKKS).^7^ When the level of C1-INH is decreased or the protein is dysfunctional, these systems are released from inhibition. The overactivation of the PKKS leads to excess bradykinin (BK) formation from high molecular weight kininogen in the plasma.^8^ BK through bradykinin B2 receptors leads to increa-sed vascular permeability, thus causing edema formation in the patients.^9^^,^^10^

Hypersensitivity is defined by the World Allergy Organization's Nomenclature Review Committee as “objectively reproducible symptoms or signs initiated by exposure to a defined stimulus at a dose tolerated by normal persons” and allergy as “a hypersensitivity reaction initiated by specific immunologic mechanisms”, which can be divided into IgE-mediated allergy (Type I) or non-IgE-mediated allergy (Types II, III, and IV in Gell-Coombs's classification).^11^ They can manifest with a broad spectrum of clinical symptoms, depending on the affected organs, and can cause mild to life-threatening symptoms. Hypersensitivity reactions in the respiratory system can lead to (allergic) rhinitis or (allergic) asthma bronchiale. The involvement of the eye can lead to (allergic) (rhino)conjunctivitis. Food hypersensitivity can manifest with gastrointestinal symptoms, but it usually causes extraintestinal symptoms as well. Skin symptoms can occur after direct contact with the provoking factor or as a systemic reaction after non-skin exposure. An anaphylactic reaction with hypotension, dyspnea, and fatigue can be life-threatening.^12^, ^13^, ^14^

In C1-INH-HAE, the deficiency of C1-INH can lead to the over-activation of the complement system. Complement plays an important role in all types of hypersensitivity reactions. Complement components, for example, anaphylatoxins C3a and C5a, can activate mast cells in Type I hypersensitivity reactions. Thus, complement activation may synergize with classical IgE-mediated responses.^15^ In types II and III, immune-complex-mediated complement activation is a key point of the pathomechanism.^16^ In Type IV, complement compo-nent C5a is a chemoattractant for T-cells and macrophages, recruiting them to the site of inflammation.^17^ On the other hand, during the degranulation of mast cells, heparin is also released amongst other substances. Heparin can activate the PKKS, leading to the generation of BK, the same molecule that is known to be responsible for edema formation in HAE patients.^18^, ^19^, ^20^, ^21^

Based on these potential links in the pathomechanism of the 2 diseases, we aimed to investigate the occurrence of different types of hypersensitivity reactions in our Hungarian patients with HAE due to C1-INH-deficiency (C1-INH-HAE). In our study, besides assessing the prevalence of hypersensitivity reactions in general, our aim was to investigate the occurrence of different types of hypersensitivity in our patients as well as to examine the connection between HAE and hypersensitivity symptoms. When we started data collection, no similar survey was present in the literature. In the meantime, in 2022, a Swedish group published the results of their survey of 239 C1-INH-HAE patients. Investigating the comorbidities, they found that the prevalence of registered allergy, asthma, or atopic dermatitis was 2 times higher in their C1-INH-HAE patients than in the general Swedish population.^22^

## Methods

### Participants and study design

We conducted a retrospective survey focusing on Hungarian C1-INH-HAE patients' hypersensitivity and HAE symptoms. The studied population consisted of diagnosed adult C1-INH-HAE patients who were monitored at the Hungarian Angioedema Center of Reference and Excellence (HU-ACARE). The diagnosis of C1-INH-HAE was established by the HU-ACARE's doctors 5 months to 44 years (median 21.4 years, Q1-Q3 13.2–30.2 years) prior to the survey by complement studies. We compiled a questionnaire of 112 questions that the participants could fill out either online (using Google Forms) or on paper. The participants were informed that the survey was voluntary and that their choice to participate or not would not affect their treatment (License number: 1067–5/2018/EÜIG). The study protocol was approved by the institutional review board, and informed consent was obtained from the participants in accordance with the Declaration of Helsinki.

We asked 156 individuals between November 29, 2021, and March 1, 2022. Out of the 106 responders, 97 patients had type I, and 9 patients had type II C1-INH-HAE. The control group was the Hungarian adult population, based on published data from several Hungarian research groups and on the results of the 2019 European Health Examination Survey (EHES) published on the Eurostat website.^23^ In the questionnaire of the EHES, 1 self-reported question on any kind of allergic disease (allergic rhinitis, eczema, food allergy, or other allergy) was present.

### Questionnaire

The questionnaire (Supplemental Appendix 1) was in Hungarian and was divided into 6 sections. In the first section, we asked general questions about different types of hypersensitivity. In the second section, questions about specific hypersensitivity symptoms were asked, while in the third part, the presentation of different types of hypersensitivity reactions was inquired. The fourth part asked questions about the presentation of HAE symptoms, while the fifth investigated the connection between HAE and hypersensitivity symptoms. In the last section, we asked questions about general data.

In our experience, patients don't understand the term “hypersensitivity” or tend to use the terms “allergy” and “hypersensitivity” as synonyms, usually preferring the use of “allergy”. Thus, to make it more understandable for our patients, in our questionnaire we used the term “allergy”.

### Data correction

As a rule of thumb, we accepted all patient-reported symptoms as hypersensitivity, as we believe that they have only reported connections that either they could observe multiple times or that were considered by medical staff as allergies. However, medical professionals at our HU-ACARE have done a brief first analysis of the data based on the detailed answers of the patients. During this first analysis, we ruled out 2 patient-reported hypersensitivity reactions. Each exclusion and the reasons for them are shown in Table 1.

**Table 1:** Patients whose reported hypersensitivities were excluded during data correction

Type of reported hypersensitivity	Provoking factor	Symptoms	Reason for exclusion
Drug hypersensitivity	Doxycycline	Photosensitivity	Known side-effect of the drug
Contact sensitivity	Cat, dog	Edema lasting 2–3 days after scratch	Most probably animal dander allergy

### Statistical analysis

Our dataset mostly consisted of categorical variables that we characterized with percentage distribution. The only exceptions were the patients’ age and the time from the diagnosis of HAE, which were characterized by median, minimal, and maximal values and lower and upper quartile values. To determine if there was a connection between variables, a chi-squared test was used. All calculations were made using Microsoft Excel 2016 and GraphPad Prism 7.0 programs, and the significance level was set at 0.05.

## Results

### Demographical data

As filling out the questionnaire was voluntary, 106 patients responded in total, amongst them 64 women and 42 men. Their median age was 46 years (min.-max. 18–90, Q1-Q3 34.75–58). Regarding place of residency, 20.8% (22/106) of the respondents lived in the capital, 19.8% (21/106) at county seats, 28.3% (30/106) in other cities, and 31.1% (33/106) in villages. Comparing these data to the whole Hungarian adult C1-INH-HAE population, no statistical difference was found in terms of sex, age, or place of residency (*p*-values 0.52, 0.79, and 0.68, respectively).

### Prevalence of hypersensitivity reactions

**Fig. 1:**
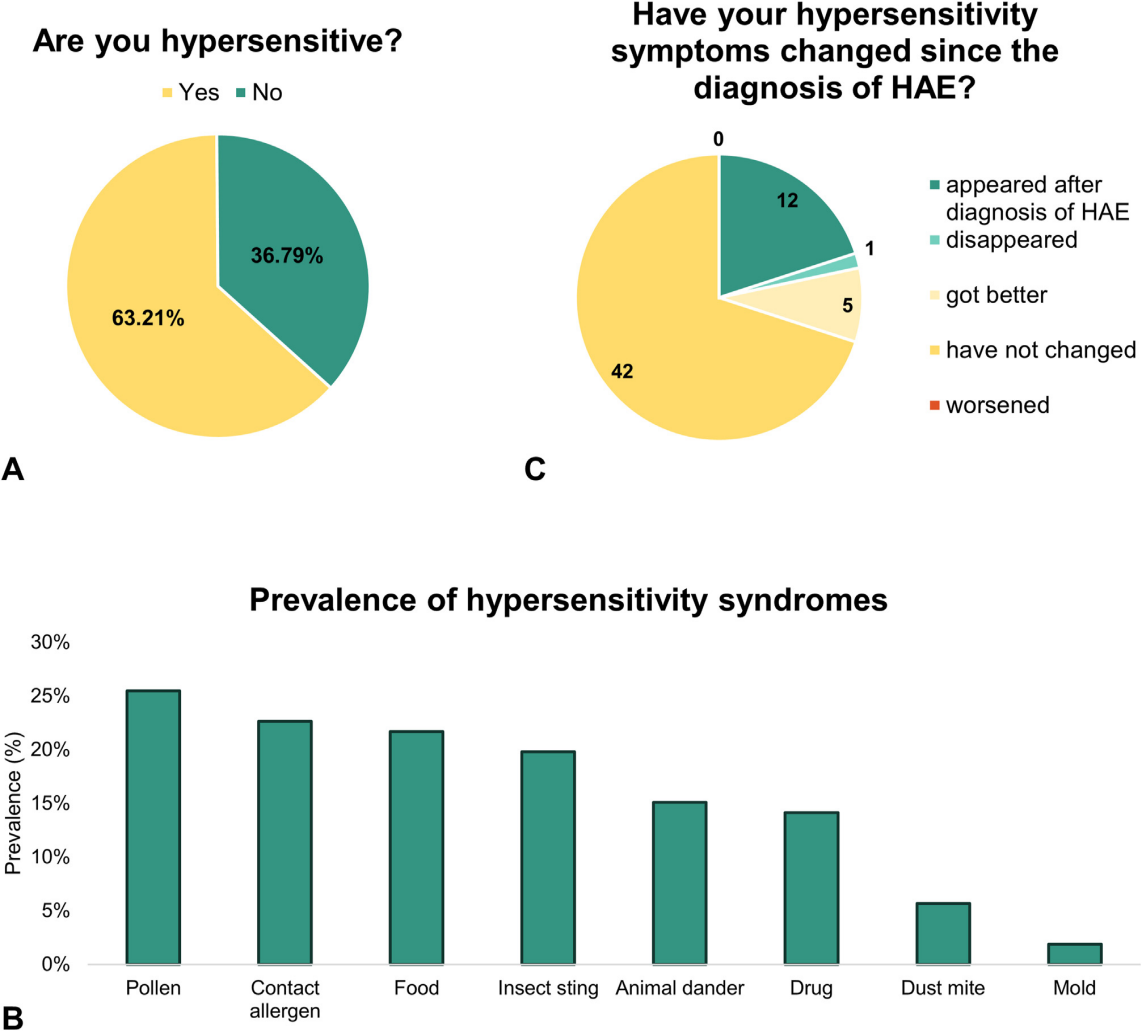
**Diagrams showing the prevalence of hypersensitivity reactions**. A, Cumulative prevalence of hypersensitivity reactions. B, Prevalence of hypersensitivity reactions. C, Change in hypersensitivity symptoms after the diagnosis of C1-INH-HAE. *Abbreviations: C1-INH-HAE – hereditary angioedema due to C1-inhibitor-deficiency; HAE – hereditary angioedema*.

Some type of hypersensitivity symptom was reported by 63.2% of the responders (67/106) where a patient could, of course, have multiple hypersensitivities (Fig. 1A). In the study, we categorized hypersensitivity reactions based on provoking factors. In Fig. 1B, we summarized the prevalence of hypersensitivity reactions. Out of the 67 hypersensitive patients, 32 (47.8%) have reported 1 type of hypersensitivity, while 35 patients (52.2%) reported more than 1. The prevalence of different hypersensitivity reactions was as follows: 25.5% (27/106) reported pollen hypersensitivity, 22.6% (24/106) contact sensitivity, 21.7% (23/106) food hypersensitivity, 19.8% (21/106) insect sting hypersensitivity, 15.1% (16/106) animal dander hypersensitivity, 14.2% (15/106) drug hypersensitivity, 5.7% (6/106) dust mite hypersensitivity, and 1.9% (2/106) mold hypersensitivity. The causes (if applicable) and symptoms are shown in Table 2.

**Table 2:** Provoking factors and symptoms of hypersensitivity reactions in Hungarian patients with C1-INH-HAE

Type of hypersensitivity	Number of patients	Provoking factors (number of patients)	Symptoms (number of patients)
Pollen hypersensitivity	27	No specific questions were asked	No specific questions were asked
Contact sensitivity	24	Metal (10) Detergent, rinsing (9) Cosmetics (7) Latex gloves (2) Balsam of Peru (1) Cement (1) Chemicals (1) Diluent (1) Disinfecting cloth (1) Hydrocolloid (1) Oil (1) Paint (1)	Itching (16) Erythema (13) Urticaria (8) Eczema (7) Edema (5) Rash (1)
Food hypersensitivity	23	Dairy products (7) Gluten (6) Nuts (5) Tomato (4) Walnut (3) Garlic (3) Coffee (2) Mushroom (2) Onion (2) Crab (1) Egg (1) Fish (1) Fructose (1) Honey (1) Kiwi (1) Orange (1) Peach (1) Poppy seed (1) Red food coloring (1) Sesame (1) Sheep curd (1) Smoked food (1) Sodium benzoate (1) Soy (1) Strawberry (1)	Bloating (9) Abdominal pain (5) Subcutaneous or submucosal edema (4) Diarrhea (3) Itching of the skin (3) Itching of the throat (3) Erythema (2) Rash (2) Dysphagia (1) Fainting (1) Rhinorrhea (1)
Insect sting hypersensitivity	21	Bee (9) Mosquito (9) Wasp (8) Horse fly (1) Spider (1)	Edema (19) Erythema (12) Itching (6) Burning sensation (2)
Pet hypersensitivity	16	Cat (10) Dog (8) Horse (3) Fish (1)	Sneezing (7) Itching of the eye (6) Itching of the skin (4) Nasal congestion (4) Rhinorrhea (4) Erythema (2) Itching of the throat (2) Tearing up (2) Blister (1) Dyspnea (1) Itching of the nose (1)
Drug hypersensitivity	15	β-lactam antibiotics (10) Other antibiotics (6) NSAIDs (2) Alprazolam (1) Dequalinium chloride (1) Glucosamine sulfate (1) Sumatriptan (1)	Rash, including urticaria (9) Itching (4) Dyspnea (3) Edema (2) Abdominal pain (1) Anxiety (1) Cough (1) Diarrhea (1) Dysphagia (1) Heat wave (1) Laryngeal edema (1) Palpitation (1) “Skin symptoms” (1) Vertigo (1)
Dust mite hypersensitivity	6	Not applicable	No specific questions were asked
Mold hypersensitivity	2	Not applicable	No specific questions were asked

Abbreviations: NSAID – Non-steroid anti-inflammatory drug.

Amongst the hypersensitive patients, 39/67 (58.2%) were female and 28/67 (41.8%) were male. Investigating the connection between the prevalence of hypersensitivity and the type of C1-INH-HAE, we found that out of our 97 patients with type I C1-INH-HAE, 61 had some type of hypersensitivity (62.9%), while this ratio was 66.6% (6/9) in the patients with type II C1-INH-HAE. There was no significant difference between the 2 groups (*p* = 0.822).

### Prevalence of provoked HAE attacks

In the questionnaire, we specifically asked about HAE attacks triggered by drugs or food. Drugs triggered HAE attacks in 11.3% of the respondents (12/106), while 23.1% (24/106) have experienced HAE attacks provoked by food. The provoking drugs and food with the provoked symptoms are shown in Table 3, Table 4, respectively.

**Table 3:** Drugs provoking HAE attacks

Patient	Drug	HAE symptoms	Drug hypersensitivity?
23 years old female	Paracetamol	SC edema	No
35 years old female	Oral contraceptive	GI edema, pain	No
39 years old female	Ciprofloxacin	GI edema	Yes *(amoxicillin + clavulanic acid)*
41 years old female	Oral contraceptive	GI edema	No
43 years old female	Fluconazole, acetylsalicylic acid	SC edema, erythema	No
49 years old female	Tramadol	SC edema, burning sensation	No
51 years old female	ACE-inhibitor (perindopril)	Edema of the lip	Yes *(penicillin, trimethoprim/sulfamethoxazole, cefuroxime, alprazolam)*
51 years old female	Simeticone	GI edema	Yes *(penicillin)*
58 years old female	Penamecillin	Abdominal pain	Yes *(penamecillin)*
77 years old female	ACE-inhibitor (captopril)	Facial edema	No
35 years old male	Antibiotic (could not name)	Abdominal pain	No
37 years old male	ACE-inhibitor (perindopril)	SC edema	No

Abbreviations: ACE – angiotensin converting enzyme; HAE – hereditary angioedema; GI – gastrointestinal; SC – subcutaneous.

**Table 4:** Food provoking HAE attacks

Patient	Food	HAE symptoms	Food hypersensitivity?
23 years old female	Coffee, alcohol, milk	SC edema	No
33 years old female	Spicy or fatty food	Abdominal pain	No
33 years old female	Strawberry, nuts, walnut	SC edema	No
35 years old female	Very fatty food	GI edema	Yes *(orange, strawberry, onion, fructose)*
35 years old female	Shallots	Abdominal pain	No
37 years old female	Gluten	GI edema	Yes *(gluten, milk)*
41 years old female	Beans, cucumber	GI edema	No
43 years old female	Garlic, mushroom	GI edema, vomiting	Yes *(mushroom)*
43 years old female	Orange, nuts, walnut	SC edema, erythema	No
48 years old female	Walnut, smoked food, sesame	GI edema	Yes *(walnut, garlic, smoked food, soy, sesame)*
48 years old female	Nuts, onion, garlic, mushroom	Abdominal pain, malaise	Yes *(kiwi, sheep curd)*
50 years old female	Orange, walnut, onion	Burning sensation in the mouth, then GI edema	No
51 years old female	Nuts, walnut, onion, garlic, purple onion, sunflower seeds	Abdominal pain	No
55 years old female	Tomato, strawberry, nuts, walnut, onion, garlic, mushroom, paprika, kiwi, pomegranate	SC edema, erythema	No
58 years old female	Nuts, onion	GI edema	No
62 years old female	Walnut, mushroom, gluten	Headache^a^	No
65 years old female	Onion	Abdominal pain, vomiting	No
75 years old female	Onion	Bloating	No
29 years old male	Leek, camembert	GI edema	No
35 years old male	Dill, fatty food	Abdominal pain	Yes *(gluten)*
35 years old male	Onion	Bloating, vomiting, diarrhea	No
36 years old male	Tomato, onion, garlic, corn, milk	GI edema	Yes *(tomato, onion, garlic, milk)*
37 years old male	Nuts, coffee, cola	GI edema	Yes *(nuts, gluten)*
58 years old male	Dairy products	GI edema	No

Abbreviations: HAE – hereditary angioedema; GI – gastrointestinal; SC – subcutaneous.

a There are data in the literature^40^, ^41^, ^42^ listing headaches as a possible, although rare, symptom of HAE attacks. We also have some patients with headaches who did not react to analgesics but were relieved after admission of C1-inhibitor. Therefore, we have accepted this patient's symptoms as an HAE attack caused by these foods.

We further investigated the connection between hypersensitivity and HAE symptoms triggered by drugs or food. Out of our 15 drug-hypersensitive patients, 4 (26.7%) have had HAE attacks provoked by drugs (Table 3). However, only 1 of them has reported the same provoking factor for HAE attack and hypersensitivity. Out of our 23 food-hypersensitive patients, 8 (34.8%) have had HAE attacks provoked by food (Table 4). Five have had the same provoking factors for both diseases, while the other 3 have had different provoking factors.

We were also interested in whether pollen-hypersensitive patients experienced more frequent HAE attacks during the “pollen season”, that we defined as the timespan from March to October. To answer this question, we looked up the symptom diary of the patients from 2017 to 2021. We calculated the monthly attack rate during the “winter season” (January–February, November–December) and the “pollen season” (March–October). We calculated the ratio of the monthly attack rates in “pollen season” and “winter season” and considered a ratio greater than 1 as a “more frequent attack rate”. We had 75 patients who filled out the questionnaire, and we also had data about their attacks available from at least 3 years prior to our interviews. Amongst these patients, 38.7% (29/75) had more frequent attacks during “pollen season”. This ratio was 45.0% (9/20) in the pollen-hypersensitive subgroup and 36.4% (20/55) in the non-pollen-hypersensitive subgroup. No statistical difference was found between the 2 groups in terms of HAE attack frequency in the pollen season (*p* = 0.497).

### Connection between HAE and hypersensitivity

To the question *“How did your allergic symptoms change after the diagnosis of HAE?“*, 60 patients chose from the options *“disappeared”*; *“got better”*; *“did not change”*; *“worsened”*; or *“the allergy presented after the diagnosis of HAE”*. Out of them, 20.0% (12/60) experienced the appearance of hypersensitivity symptoms after the diagnosis of HAE. The hypersensitivity symptoms disa-ppeared in 1.7% (1/60), got better in 8.3% (5/60), did not change in 70.0% (42/60), and no worsening of the hypersensitivity symptoms was experienced after the diagnosis of HAE by any of the patients (Fig. 1C). Out of the 6 patients whose hypersensitivity symptoms improved (disappe-ared or got better) after diagnosis, 3 received prophylactic HAE treatment (50.0 %). Amongst the 42 patients who did not experience any chan-ge in their hypersensitivity symptoms, 9 (21.4 %) were on prophylaxis (Table 5).

**Table 5:** Change of hypersensitivity symptoms in patients with different types of prophylactic treatment.

After the diagnosis of HAE, the hypersensitivity symptoms…	No prophylactic treatment	Continuous danazol	Other prophylactic treatment	Altogether
… appeared	10	2	0	12
… disappeared	0	0	1 (danazol, then clinical study)	1
… got better	3	2	0	5
… did not change	33	9	0	42
… worsened	0	0	0	0

Abbreviations: HAE – hereditary angioedema.

The questions about the connection between HAE and hypersensitivity symptoms were answered by 51 patients. The appearance of HAE attacks worsened the hypersensitivity symptoms of 7.8% (4/51). Twice as many, 15.7% (8/51) have experienced at least 1 HAE attack triggered by hypersensitivity. The hypersensitivities and the provoked HAE attacks can be seen in Table 6.

**Table 6:** HAE attacks provoked by hypersensitivity

Patient	Hypersensitivity	Provoked HAE attack
33 years old female	Pollen and contact sensitivity	SC edema
48 years old female	Pet hypersensitivity Food hypersensitivity	Scratch by the animal caused irritation and edema of the skin GI edema
48 years old female	Pollen hypersensitivity Food hypersensitivity	Dyspnea Abdominal problems
50 years old female	Drug hypersensitivity	GI edema
58 years old female	Drug hypersensitivity	Abdominal pain
58 years old female	Pollen, pet, and contact sensitivity	Abdominal pain
62 years old female	Food and pet hypersensitivity	“Mottling” *(probably erythema marginatum)*
37 years old male	Gluten sensitivity	GI edema after consuming gluten

Abbreviations: HAE – hereditary angioedema; GI – gastrointestinal; SC – subcutaneous.

## Discussion

First, we confirmed that the responders were representative of the Hungarian adult C1-INH-HAE population, as no significant connection could be observed between the group (study population or Hungarian C1-INH-HAE patients) and sex, age, or place of residency. In our C1-INH-HAE patient group, the prevalence of hypersensitivity reactions was rather high, with pollen hypersensitivity being the most prevalent form.

In 2016, a questionnaire-based survey investigated ragweed allergy in 1000 Hungarian adults. Thirty-one percent of their respondents have had allergic rhinitis, and 22% of the respondents experienced allergic rhinitis during “ragweed season” (between August and September).^24^ Our results are comparable with these findings, as we detected a 25.5% prevalence of pollen hypersensitivity. However, in 2019, another survey was conducted in schoolchildren regarding allergic rhinitis in Budapest, the capital of Hungary, where the parent-reported prevalence of allergic rhinitis was found to be 29.3%, while only 9.7% of the children were diagnosed by a physician.^25^ This suggests a higher perceived prevalence if we rely on questionnaires instead of clinical diagnoses, which can be true about our survey and that of Márk et al^24^ as well.

The second most common type of hypersensitivity in our patients was contact sensitivity. Between 1998 and 1999, a multi-center study was conducted in Hungary to assess the prevalence of fragrance contact sensitization. In this study, 8.2% of patients reacted to the fragrance mix skin test.^26^ Between 2007 and 2014, 3631 patients were patch tested in a Hungarian dermatology center to determine the prevalence of *para*-phenylenediamine sensitivity. In this population, 5.76% were sensitive to this potent allergen found in hair dyes and henna tattoos.^27^ In 2014, methylisothiazolinone (MI) sensitivity was investigated in 314 patients in a Hungarian dermatology center. Eight percent were found to be allergic to MI, a common ingredient in cosmetics at the time of the survey.^28^ Between 2013 and 2014, the occurrence of lavender oil hypersensitivity was studied in 7 Hungarian dermatological centers. Lavender oil hypersensitivity was found in 0.53% of the 1509 patients investigated.^29^ In our C1-INH-HAE patients, the prevalence of contact sensitivity was 22.6%. It seems higher than the ratios mentioned above, but we must emphasize that we did not differentiate between types of contact sensitivity, whereas the previous articles all investigated special types. Moreover, the above-mentioned bias due to self-report can also contribute to the observed higher prevalence. Nevertheless, in our population, 6.6% were hypersensitive to cosmetics. As MI might be 1 of the substances causing allergy in cosmetics, our data was similar to that of Pónyai et al.^28^

Food hypersensitivity was also quite frequently reported in our C1-INH-HAE patients. In 2020, a survey about food allergies was conducted in a Hungarian center on 501 adults. Intolerance to biogenic amines was found in 50%, oral allergy syndrome was confirmed in 14%, and 1% was diagnosed with IgE-mediated food allergy. The most frequent provoking factors were fruits (40%), milk or dairy products (35%), and nuts or oilseeds (29%).^30^ In our patient group, 21.7% reported food hypersensitivity, with dairy products (30.4%), gluten (26.1%), nuts (21.7%), and tomatoes (17.4%) being the most frequent provoking factors.

To the best of our knowledge, no comprehensive data has been published in the last 20 years regarding the occurrence of allergy in the Hungarian adult population caused by animal dander, drugs, insect sting, dust mite, or mold. One-fifth of our patients reported insect sting hypersensitivity, with bees, mosquitos and wasps being the most frequent provoking factors and edema and erythema being the most frequent symptoms. We found that approximately one-seventh of our C1-INH-HAE patients had animal dander hypersensitivity, with cats and dogs being the most frequent provoking factors and sneezing and itching being the most frequent symptoms. Similarly, one-seventh of our patients had drug hypersensitivity, with β-lactam and other kinds of antibiotics being the most frequent provoking factors and rash being the most frequent symptom. One-twentieth of our patients had dust mite hypersensitivity, and we also had 2 patients who had mold hypersensitivity.

One-tenth of our patients has experienced an HAE attack provoked by drugs (Table 3). To the best of our knowledge, no antibiotic-associated HAE attack has been described in the literature. A possible explanation is mentioned amongst the limitations of our study (limitation d). Angiotensin-converting enzyme (ACE) inhibitors are known triggers of bradykinin-mediated edema^31^, ^32^, ^33^ as ACE is 1 of the most important bradykinin-degrading enzymes.^8^^,^^34^ Estrogen, a component of oral contraceptives, is also a known trigger factor for HAE attacks, most probably through a FXII-mediated pathway.^35^, ^36^, ^37^

One-fifth of our patients has experienced an HAE attack provoked by food (Table 4). Our findings are in line with the results of a Swiss group that also found that the most common food triggers for HAE attacks were onions and dairy products, causing abdominal edema.^38^

Regarding the relationship between C1-INH-HAE and hypersensitivity, we found only 1 patient with a drug that caused both and 5 patients with the same food causing both. In addition, we could not identify a statistical connection between pollen hypersensitivity and the HAE attack rate during the pollen season. Approximately one-sixth of hypersensitive patients have experienced HAE attacks provoked by a hypersensitivity reaction. These findings suggest that there is no strong statistical connection between hypersensitivity reactions and HAE attacks. However, on the level of individual patients, a connection between hypersensitivity and HAE symptoms could be observed. As the course of C1-INH-HAE shows high intra- and inter-individual variability,^3^^,^^4^ we emphasize the importance of individualized treatment plans. The number of patients examined was also quite low, so further studies with more patients are needed.

Most of our patients have not experienced any change in their hypersensitivity symptoms after the diagnosis of HAE, and those who felt their hypersensitivity symptoms had changed all reported milder symptoms of hypersensitivity after the diagnosis of HAE. We could think of 3 possible explanations: (a) after being diagnosed with HAE, the patients can get adequate therapy for their C1-INH-HAE symptoms, thus reducing stress levels, which leads to a more balanced lifestyle with less severe hypersensitivity symptoms; (b) after the diagnosis of C1-INH-HAE, the patients are referred to an allergologist-immunologist, who, aside from treating their HAE, can provide them with treatment for their hypersensitivity as well; and (c) prior to the diagnosis of C1-INH-HAE, edematous symptoms might have been misdiagnosed as allergy. However, a high rate of hypersensitive C1-INH-HAE patients reported that their hypersensitivity symptoms had started after the diagnosis of HAE, suggesting that the treatment of C1-INH-HAE by itself is not enough to prevent hypersensitivity reactions.

The last EHES was carried out in 2019 in Hungary. In the questionnaire, 1 self-reported question on any kind of allergic disease was present. The result of the survey displayed on the Eurostat website reported the prevalence of allergy in the Hungarian population to be 19.3%.^23^ In contrast, in our C1-INH-HAE patient population, the ratio of reported hypersensitivity was 63.2%. Since the methods of the 2 studies (self-reported questionnaires, asking about allergic diseases) were similar, these findings suggest a connection between the 2 diseases. An interesting phenomenon is that although most of our findings are in line with surveys of the Hungarian adult population, the number of hypersensitive patients is 3 times higher in our C1-INH-HAE patients than in the Hungarian population. We can think of 4 possible explanations: (a) The bias could be explained by a higher prevalence amongst our patients of the types of hypersensitivity reactions that have not yet been investigated in the Hungarian population (animal dander, drug, insect sting, dust mite, or mold hypersensitivity). However, the prevalence of these diseases is 15% or lower in our patients, so it is unlikely that this phenomenon could cause a threefold growth; (b) It could be possible (although unlikely) that amongst our patients, there are more with only 1 type of hypersensitivity reaction than in the general population; (c) The surveys examining the prevalence of specific allergic diseases were conducted in allergology centers, and most of the examined people were patients at these centers; thus, allergic diseases are likely to be overrepresented in these surveys, just like in our study based on self-reported answers; and (d) For some reason, the allergic persons were underrepresented in the EHES.

In 2022, a survey was conducted in Sweden, where the prevalence of registered allergy, asthma, or atopic dermatitis was found to be 2 times higher in the C1-INH-HAE population than in the general Swedish population.^22^ Our results are in line with this finding.

We found no significant difference in the prevalence of hypersensitivity reactions between our type I and type II C1-INH-HAE patients, which is reasonable, as the suggested common pathways of C1-INH-HAE and hypersensitivity through over-activation of the complement system and through heparin release are similarly affected in both cases.

However, our study has its limitations. (a) As the questionnaire was self-reported, we had to rely on the patients' own definition of hypersensitivity, hypersensitivity symptoms, and HAE attacks; therefore, the data obtained this way might be biased by patients' different interpretation of symptoms. (b) As the filling of the questionnaire was voluntary, there is a chance that those who have hypersensitivity were more likely to respond than those who do not, potentially raising the perceived prevalence of hypersensitivity. (c) C1-INH-HAE is a rare disease with a prevalence of 1:50 000.^39^ In consequence, the number of patients involved in the survey is rather low. (d) When talking about hypersensitivity reactions and HAE attacks provoked by drugs, in this study, we relied on patients’ reports and did not investigate potential underlying mechanisms. For example, when an antibiotic was mentioned as the provoking factor, there had to have been an underlying infection that required treatment with antibiotics. The occurring symptoms (rash, abdominal pain) might have been caused by the disease itself as well as by the antibiotic treatment. In this study, however, we treated all reported cases as hypersensitivity reactions or drug-provoked HAE attacks as reported by the patients.

To conclude, we investigated the occurrence of hypersensitivity reactions amongst Hungarian C1-INH-HAE patients followed at our HU-ACARE based on potential links in the pathomechanism of the 2 diseases. We found that the prevalence of hypersensitivity reactions in the C1-INH-HAE population is approximately 3 times higher than in the general Hungarian population, suggesting a connection between the 2 diseases. However, this study was only the first step of our research investigating the connection between allergy and C1-INH-HAE. Here, our aim was to investigate the clinical manifestations of hypersensitivity symptoms in our patients. We are aware that the information collected this way is biased and cannot be used to diagnose allergies in our patients. Therefore, we have planned and already launched the second part of our study, with 2 aims in mind. First, we would like to examine the prevalence of allergic diseases in our patients with the help of their previous medical records and blood samples collected in our biobank. Secondly, we are planning to measure selected mast cell mediators in the blood samples collected from our patients and to compare allergic and non-allergic HAE-patients’ samples.

## Abbreviations

ACE, Angiotensin-converting enzyme; BK, Bradykinin; C1-INH, C1-inhibitor protein; C1-INH-HAE, Hereditary angioedema due to C1-inhibitor deficiency; EHES, European Health Examination Survey; FXII, Coagulation factor XII; HAE, Hereditary angioedema; HU-ACARE, Hungarian Angioedema Center of Reference and Excellence; MI, Methylisothiazolinone; PKKS, Plasma kinin-kallikrein system.

## Funding

This work was supported by the 10.13039/501100015269Hungarian Government and the 10.13039/501100000780European Union [grant number EFOP-3.6.3-VEKOP-16-2017-00009]; and the Hungarian National Research, Development And Innovation Office [grant number NKFI 124557]. The funding providers had no role in the research and/or the preparation of the manuscript.

## Availability of data and materials

The datasets generated during and/or analyzed during the current study are available from the corresponding author on reasonable request.

## Author contributions

Concept and design: Hanga Réka HORVÁTH, Zsuzsanna BALLA, Henriette FARKAS.

Collection of data: Hanga Réka HORVÁTH, Dávid SZILÁGYI, Beáta VISY, Henriette FARKAS.

Data analysis: Hanga Réka HORVÁTH, Noémi ANDRÁSI, Zsuzsanna BALLA, Beáta VISY, Henriette FARKAS.

Manuscript writing: All authors.

Final approval of manuscript: All authors.

## Ethics statement

The study protocol was approved by the institutional review board of Semmelweis University, Budapest, and informed consent was obtained from the participants in accordance with the Declaration of Helsinki. Before completing the survey, the participants were informed that the survey was voluntary and that their choice to participate or not would not affect their treatment (Licence number: 1067–5/2018/EÜIG).

## Authors’ consent for publication

All authors give their consent to the publication of this work.

## Submission declaration

The authors hereby declare that this manuscript has not been published previously (except in the form of an abstract and academic thesis), that it is not under consideration for publication elsewhere, that its publication is approved by all authors and tacitly by the responsible authorities, and that, if accepted, it will not be published elsewhere in the same form, in English or in any other language, including electronically without the written consent of the copyright holder.

## Declaration of competing interest

Hanga Réka HORVÁTH has received travel grants from Takeda.

Dávid SZILÁGYI has no conflict of interest to declare.

Noémi ANDRÁSI has no conflict of interest to declare.

Zsuzsanna BALLA has participated in clinical trials of CSL Behring, Pharvaris and Takeda.

Beáta VISY has participated in clinical trials of Kalvista, CSL Behring, Pharvaris and Takeda.

Henriette FARKAS has received research grants from CSL Behring, Takeda and Pharming and served as an advisor for these companies and Kalvista and Biocryst, and has participated in clinical trials/registries for BioCryst, CSL Behring, Pharming, Kalvista, Pharvaris and Takeda.

## Appendix A Supplementary data

Multimedia component 1 Translated version of the ”Investigating allergic diseases in patients with hereditary angioedema due to C1-inhibitor deficiency” questionnaire used for data collection. Multimedia component 1
