# Supplementary material for: Hypersensitivity reactions amongst Hungarian Patients with Hereditary Angioedema due to C1-Inhibitor Deficiency
Source: World Allergy Organ J. 2023 Oct 23;16(11):100833. doi: 10.1016/j.waojou.2023.100833 (PMC10618765; doi:10.1016/j.waojou.2023.100833)
Supplement: Multimedia component 1 — Translated version of the ”Investigating allergic diseases in patients with hereditary angioedema due to C1-inhibitor deficiency” questionnaire used for data collection. [file mmc1.pdf]

”Investigating allergic diseases in patients with hereditary angioedema due to C1-inhibitor deficiency” questionnaire

Do you have any allergic diseases?...yes/no

If yes, what kind of allergic disease do you have?

urticaria / eczema / pollen sensitivity / food allergy/ drug allergy / pet allergy /

/ contact allergy / insect sting allergy / other: _______________________________

If yes, for how many years have you had your symptoms? ___________________________

Questions regarding allergic symptoms:

To make our survey more precise, we ask detailed questions about the potential allergic diseases:

Have you experienced allergic reactions on your skin? ....yes / no

If yes, what symptoms did you experience?

urticaria / eczema / itching / redness / swelling / other: _________________

If yes, in what localisation?

face, head / neck / trunk / bends / extremities

Have you experienced allergic reactions in your airways? ....yes / no

If yes, what symptoms did you experience?

coughing / dyspnoea / whistling / asphyxia / other: ____________________

Have you experienced allergic rhinitis? ....yes / no

If yes, what symptoms did you experience?

nasal congestion / runny nose / itching nose / sneezing / other: ___________

Have you experienced allergic symptoms in your eyes?...yes / no

If yes, what symptoms did you experience?

itching / discharge / tearing up / redness / edema of the eye / other: _______

Have you experienced allergic symptoms in your intestines?...yes / no

If yes, what symptoms did you experience?

bloating / diarrhoea / abdominal pain / other: ___________________________

Have you experienced other allergic symptoms?..yes / no

If yes, what symptoms did you experience?

itchy throat / loss of hearing / other: ___________________________________

Questions regarding occurrence of allergic symptoms:

How serious are your symptoms throughout the year? Please indicate in the table below, where:

0: no symptoms; 1: very mild symptoms; 2: mild symptoms; 3: average symptoms; 4: strong symptoms; 5: very strong symptoms

January	0	1	2	3	4	5	Cannot determine
February	0	1	2	3	4	5	Cannot determine
March	0	1	2	3	4	5	Cannot determine
April	0	1	2	3	4	5	Cannot determine
May	0	1	2	3	4	5	Cannot determine
June	0	1	2	3	4	5	Cannot determine
July	0	1	2	3	4	5	Cannot determine
August	0	1	2	3	4	5	Cannot determine
September	0	1	2	3	4	5	Cannot determine
October	0	1	2	3	4	5	Cannot determine
November	0	1	2	3	4	5	Cannot determine
December	0	1	2	3	4	5	Cannot determine

When do your allergic symptoms occur during the day?

in the morning / during the day / in the evening / at night-time / all day long /

/ Cannot determine

Do your allergic symptoms occur regularly during the day?

regularly / irregularly / Cannot decide / Cannot determine

In what environment do your allergic symptoms present themselves?

outdoors / in smog / after physical strain / at home / at work: ____________

/ other place: _________________/ Cannot determine

Have you experienced pollen sensitivity? ....yes / no

If yes, do you feel that your allergic symptoms are more frequent between March and October? .... Much more frequent / more frequent / no

Have you experienced allergic symptoms after meals?..yes / no

If yes, after consuming what types of food?

tomato / orange / strawberry / walnut / nuts / brown onion / garlic / mushroom /

/ other: ____

If yes, what symptoms did you experience? ___________________________________

Have you experienced allergic symptoms after taking a drug?.......................................yes / no

If yes, after taking what drug?____

If yes, after taking what amount of drug? ____________________________________

If yes, what symptoms did you experience? ___________________________________

Have you experienced allergic symptoms after contact with an animal?.........................yes / no

If yes, after contact with what kind of animal?

dog / cat / horse / bird / other: ____

If yes, what symptoms did you experience? ___________________________________

Have you experienced symptoms thought to be allergic after direct contact of your skin with particular materials? ....yes / no

If yes, after contact with what kind of materials?

metal (accessories, watch, buckle) / rubber gloves / cosmetics /

/ washing powder, rinsing / other:___________________________________

If yes, what symptoms did you experience?

urticaria / eczema / itching / redness / edema / other: ___________________

Have you experienced allergic symptoms after being bitten by an insect?......................yes / no

If yes, after the bite of which insect?

bee / mosquito /wasp / other:_______________________________________

If yes, what symptoms did you experience? _________________________________

Have you had skin test for allergic diseases? ....yes / no

If yes, for what reason? ____

If yes, in which year? ____

If yes, what was the result? ____

Is/was your allergy (being) treated? .... yes / no / I am not allergic

If yes, with which drug? ____

If yes, in what form do you take it?____

If yes, in what dosage do you take the medicine?_____________________________

Have your allergic symptoms changed since the diagnosis of HAE?

disappeared / got better / did not change / worsened /

/ the allergy presented after the diagnosis of HAE / I am not allergic

Questions regarding HAE:

How many HAE attacks did you have between 1 October 2020 and 30 September 2021 in these regions?

abdominal: face: neck: larynx: arms: legs: genitals:

In which region are the HAE attacks the most frequent? ________________________

Have you experienced HAE attack after taking a drug? ....yes / no

If yes, after taking what drug?____

If yes, after taking what amount of drug? ____________________________________

If yes, what symptoms did you experience? __________________________________

Have you experienced HAE attack after meals? ....yes / no

If yes, after consuming what types of food?

tomato / orange / strawberry / walnut / nuts / brown onion / garlic /

/ mushroom / other: ____

If yes, what symptoms did you experience? __________________________________

Have you experienced that your HAE attacks are more frequent between March and October?

....yes / no

Questions regarding the connection between HAE and allergic diseases:

Does the occurrence of HAE attacks worsen your allergic symptoms?

yes / no / I am not allergic

Do HAE attacks provoke allergic symptoms?.................................. yes / no / I am not allergic

If yes, what kind of symptoms?____

Do allergic symptoms provoke HAE attacks?.................................. yes / no / I am not allergic

If yes, what kind of symptoms?____

General questions:

Sex .... female / male

Age: _______________ years

Place of residence .... capital / county seat / city / village

Do you have any diseases besides HAE and allergy? ....yes / no

If yes, what kind of diseases do you have? ___________________________________

Do you have asthma?..yes / no

If yes, for how many years have you had your symptoms? ______________________

If yes, what medication do you take? _______________________________________

If yes, in what dosage do you take the medicine?______________________________

Do you regularly take some kind of medication?..yes / no

If yes, what medicine(s) do you take?_______________________________________

If yes, in what dosage do you take the medicine?______________________________

Do you smoke? ....yes / no

If yes, for how many years have you been smoking? ____________________________

If yes, how many cigarettes do you smoke daily?_______________________________

Do you smoke e-cigarettes?...yes / no

If yes, for how many years have you been smoking? ____________________________

If yes, how many cigarettes do you smoke daily?_______________________________

Did you use to smoke before? ....yes / no

If yes, for how many years did you smoke? ___________________________________

If yes, when did you quit? ____

If yes, how many cigarettes did you use to smoke daily? _________________________

Did you use to smoke e-cigarettes before?..yes / no

If yes, for how many years did you smoke? ___________________________________

If yes, when did you quit? ____

If yes, how many cigarettes did you use to smoke daily? _________________________

How often do you consume alcohol? (Please choose the option best describing your habits)

I do not drink / 2-3 times per month / 1 time per week / 3-5 times per week / daily

How many coffees do you drink a day? ____

What is your occupation? ____

Do you have any hobbies?____

Do you do sports regularly?..yes / no

If yes, what kind of sport do you do? ____

How many times do you exercise a week?

1-2 / 3-4 / 5-6 / more than 6

What is the building material of the building where you live?

panel / brick / aerated concrete / mudbrick / wood / other:_______________________

Are there any of the following present in your home? (Please circle if yes)

wall-to-wall carpet / linoleum / wallpaper / floor heating / indoor plants / water culture /

/ moldy surface / basement / feather pillow / animal hair / pet / none of the above

Is there someone else in your family who has allergic disease? ....yes / no

If yes, who is it? ____

If yes, what kind of allergy do they have?_____________________________________

Has there been in your family? (Please circle if yes)

autoimmune disease / cancer / high blood pressure / myocardial infarction / diabetes /

/ other chronic disease: ___________________________________ / none of the above
